# Capnography and Pulse Oximetry Improve Fast Track Extubation in Patients Undergoing Coronary Artery Bypass Graft Surgery: A Randomized Clinical Trial

**DOI:** 10.3389/fsurg.2022.826761

**Published:** 2022-05-11

**Authors:** Seyed Tayeb Moradian, Fatemah Beitollahi, Mohammad Saeid Ghiasi, Amir Vahedian-Azimi

**Affiliations:** ^1^ Atherosclerosis Research Center, Nursing Faculty, Baqiyatallah university of Medical Sciences, Tehran, Iran; ^2^ Atherosclerosis Research Center, Nursing Faculty, Baqiyatallah university of Medical Sciences, Tehran, Iran; ^3^ Atherosclerosis Research Center, Medicine Faculty, Baqiyatallah University of Medical Sciences, Tehran, Iran; ^4^ Trauma Research Center, Nursing Faculty, Baqiyatallah University of Medical Sciences, Tehran, Iran

**Keywords:** airway extubation, coronary artery bypass, capnography, blood gas analysis, pulse oximetry

## Abstract

**Background:**

Use of capnography as a non-invasive method during the weaning process for fast track extubation (FTE) is controversial. We conducted the present study to determine whether pulse oximetry and capnography could be utilized as alternatives to arterial blood gas (ABG) measurements in patients under mechanical ventilation (MV) following coronary artery bypass graft (CABG) surgery.

**Methods:**

In this randomized clinical trial, 70 patients, who were candidates for CABG surgery, were randomly assigned into two equal groups (*n* = 35), intervention and control group. In the intervention group, the ventilator management and weaning from MV was done using Etco2 from capnography and SpO2 from pulse oximetry. Meanwhile, in the control group, weaning was done based on ABG analysis. The length of intensive care unit (ICU) stay, time to extubation, number of manual ventilators setting changes, and alarms were compared between the groups.

**Results:**

The end-tidal carbon dioxide (ETCO2) levels in the intervention group were completely similar to the partial pressure of carbon dioxide (PaCo2) in the control group (39.5 ± 3.1 vs. 39.4 ± 4.32, *p* > 0.05). The mean extubation times were significantly shorter in the intervention group compared to those in the control patients (212.2 ± 80.6 vs. 342.7 ± 110.7, *p* < 0.001). Moreover, the number of changes in the manual ventilator setting and the number of alarms were significantly lower in the intervention group. However, the differences in the length of stay in ICU between the two groups were not significant (*p* = 0.219).

**Conclusion:**

Our results suggests that capnography can be used as an alternative to ABG. Furthermore, it is a safe and valuable monitor that could be a good alternative for ABG in this population. Further studies with larger sample sizes and on different disease states and populations are required to assess the accuracy of our findings.

**Clinical Trial Registration:**

Current Controlled Trials, IRCT, IRCT201701016778N6, Registered 3 March 2017, https://www.irct.ir/trial/7192.

## Introduction

Fast track extubation (FTE) is an accepted method for weaning patients from mechanical ventilation (MV) after coronary artery bypass graft (CABG) surgery. The duration of MV in patients undergoing cardiac surgery is usually between 1–6 h. Long-term MV might trigger certain complications, such as infection, atelectasis, and increased mortality (1, 2). With a proper management and early withdrawal of the endotracheal tube, the complications and costs would reduce (1, 3). FTE can decrease health care costs following CABG surgery up to 50% (4). Utilizing a proper anesthesia technique during surgery and post-surgery management, FTE can be applied in patients undergoing CABG surgery without any specific complications (5).

The readiness of patients for weaning is usually assessed by arterial blood gas (ABG) sampling. ABG is the gold standard for the monitoring of oxygenation and ventilation during the postoperative MV (6). Arterial line cannulation is employed in the operating room and ICU settings to provide easy access for continuous and real-time systemic blood pressure measurements, blood gas analysis, and other laboratory measurements. However, based on evidence, this process is not without risk factors (7). The process of inserting arterial line (over 48 or 72 h) is an invasive procedure with several complications, for instance infection, local hematoma, vascular injury, thrombosis, disseminated intravascular coagulation, and reduced cardiac output (8–10). On the other hand, Iran, as a developing country with sanctions, cannot access advanced medical equipment, such as ABG analyzers. Therefore, in order to minimize these complications and having easier access, finding an alternative non-invasive method seems to be reasonable.

The use of pulse oximetry as a valid non-invasive tool for oxygenation monitoring has been agreed upon by the public for many years (11). In several cases, the pulse oximetry is used as the only criterion to check oxygenation and to make oxygenation-associated changes in MV. The utilization of a capnography is also recommended under different clinical conditions (12). Capnography is a method in which the infrared radiation is employed to measure the carbon dioxide (CO2) in exhaled air. This method includes the non-invasive measurement of CO2, providing information on ventilation (effectiveness of CO2 elimination), perfusion (CO2 transportation in vasculature), and metabolism (production of CO2 via cellular metabolism) in intubated and spontaneously breathing patients (13, 14). Capnography in certain conditions, for instance confirmation of the correct place of the endotracheal tube, is recommended as the standard of care (15). Yet in some other circumstances, such as cardiopulmonary resuscitation and changing the MV settings, there are numerous differences between the existing data and there are no similar recommendations (16–18). Certain studies have mentioned the lack of correlation between these variables and have considered the difference between them high (19). On the other hand, some other studies have reported an equal role for these two parameters in monitoring the level of CO2 (14, 20).

The substantial debate is that whether capnography and pulse oximetry could be efficient alternatives for ABG analysis during the process of weaning from mechanical ventilation (MV). Therefore, capnography and pulse oximetry are used in this study for monitoring during the MV and management of the ventilator setting. The current study aimed to assess the feasibility and safety of non-invasive monitoring techniques during the weaning process in patients undergoing the CABG surgery.

## Methods

### Study Design

This study was a randomized controlled clinical trial conducted between February and March 2018 with Trial Registration Number (IRCT201701016778N6) in Iranian Registry of Clinical Trials (IRCT). The study protocol was reviewed and approved by the Ethics Committee of Baqiyatallah University of Medical Sciences, Tehran, Iran, under code IR.BMSU.REC.1395.141, in accordance with the Declaration of Helsinki of the World Medical Association (21). In addition, one day ahead of the operation, we explained the objectives of this study to the participants and obtained the informed consent. The study was conducted and reported in accordance with the recommendations of the Consolidated Standards of Reporting Trials (CONSORT) statement (22).

### Population Study

We conducted this research on 70 patients, who underwent CABG surgery at Jamaran Heart Hospital in Tehran, Iran. The patients who met the inclusion criteria were selected through convenient sampling and randomly assigned into two equal groups (*n* = 35); the intervention group and the control group. Due to the nature of this clinical trial study, which compared two different methods, there was no possibility of blinding. Randomization sequence was created with Excel 2007 (Microsoft, Redmond, WA, USA) with a 1:1 allocation, using random block sizes of 2 and 4 by an investigator with no clinical involvement in the trial. The eligible participants in this study were those with a negative history of stroke or other severe neurologic disorders, chronic obstructive pulmonary disease, and ejection fraction (EF) less than 30%. On the other hand, patients below the age of 18 or over 80, with chest tube drainage >400 mL/h at the first 4 h after surgery, hemodynamic instability (requiring intra-aortic balloon pump or high dose inotrope), loss of consciousness, and Those who were expected to require MV more than 24 h were excluded.

### Sample Size

We calculated the sample size of this study based on the Altman’s nomogram for the two-sided hypothesis with a power of 90% and α = 0.05 and according to similar studies for the weaning time variable (*p *= 0.03 and the effect size = 1.3) (23). Based on the nature of the clinical trial study and the probability of sample size drop, 10% drop was considered as the attrition rate and the final sample size for each group was considered to be 35 subjects.

### Our Center

Our center is a single specialty heart center with a multi-disciplinary team including cardiac surgeon, cardiac anesthesiologist, nurses, internal medicine, echocardiographist, perfusionist, electro-physiologist, interventionist, and cardiac rehabilitation specialist. Patients who are candidate for surgery are assessed by anesthesiologist before surgery. Also, all patients above 60 years old are screened by pulmonologist and based on comorbidities, patients are screened by nephrologist, neurologist, endocrinologist and hematologist. Our ICU is a 10-bed ICU which is designed for cardiac surgical procedures. The ICU and operation room are in the same environment and on two floors. After the surgery, patients are transferred directly to the ICU and managed by experienced nurses. Usually, patients remain in ICU for two days, and the enhanced recovery after surgery protocols, including fast tract extubation, early mobilization, and minimal sedation strategy, are utilized. Sedation infusion and also using from bolus doses of sedatives such as midazolamis discouraged and patients are oriented as soon as possible. Fast tract extubation is the routine procedure during the weaning and extubation.

### Procedure and Data Collection

**Figure 1:**
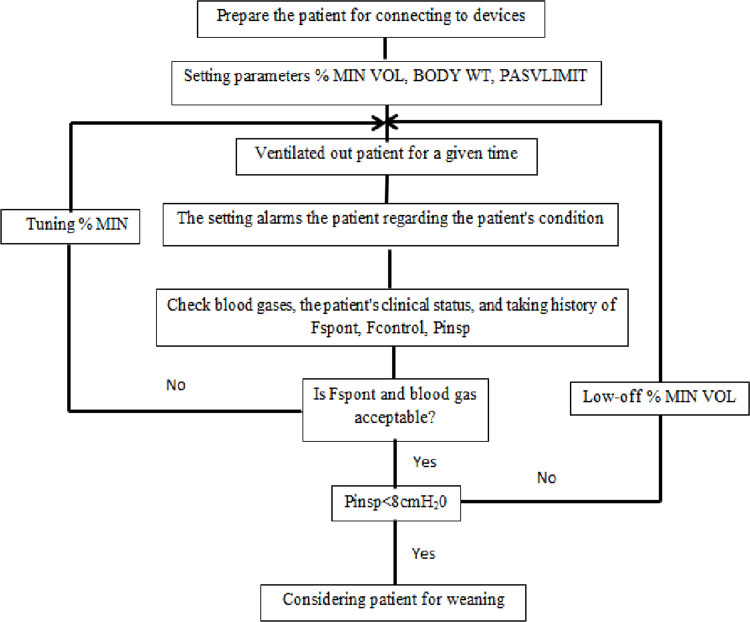
Standard weaning process algorithm in adaptive support ventilation (ASV) mode. MIN VOL, minute volume; WT, weight; F spont, frequency of spontaneous breathing; F control, frequency of controlled breathing; P insp, inspiratory pressure.

Anesthesia and surgical protocols were similar in both groups. Anesthesia was induced and maintained using midazolam, fentanyl, and propofol, and paralysis was achieved by atracurium. The patients were intubated after anesthesia induction and ventilated during the surgery. The following setting was applied in the operating room: tidal volume of 8–10 mL/kg, positive end-expiratory pressure (PEEP) of 5 cmH2O, and respiratory rate (RR) of 12 breaths per minute. During cardiopulmonary bypass, ventilation was stopped and continuous positive airway pressure of 5 cmH2O was applied. We performed the surgery using the standard procedure through median sternotomy (24). Following the surgery, adaptive support ventilation (ASV) was applied for mechanical ventilation management. A Galileo with software version GBC 01.202 (Hamilton Medical AG, Rhäzüns, Switzerland) was employed for managing the patient during the MV. Although weaning and extubation is a clinical decision made at the bedside, it is based on many factors including wakefulness, comfort, and the patient’s ability to cough and secrete. In both groups, the MV weaning criteria were MIN VOL% 60–65%, F control (number of controlled breathes) zero, f Spont >10 (number of spontaneous breathes), *p* insp <8 (the amount of automatically adjusted pressure support). Figure 1 represents standard weaning process algorithms in ASV mode (25).

In both groups, the initial ventilator settings were minute volume 100%, oxygen inspiratory fraction (Fio_2_) of 100%, positive end-expiratory pressure of 5 cm H_2_O, and flow trigger sensitivity of 4 L/min. Following 10 min, we performed an ABG analysis. Simultaneously, the ETCO2 and SPO2 were measured. The goal for PaCO2 was 38–43 mmHg. Based on our clinical pilot, the Etco2 was 2–4 mmHg lower than PaCO2. Therefore, this difference was considered in all the settings and interpretations regarding PaCO2. If the PaCO2 was greater than 45 the minute volume increased by 10%–20%, and if it was lower than 35 the minute volume decreased 10%–20%. In the intervention group, the trend was monitored with capnography and in the control group, another ABG analysis was performed 10–20 min after any changes in the setting. In the intervention group point by point changes were made in ventilation and oxygenation based on continuous monitoring of capnography and pulse oximetry. The capnography and pulse oximetry, used in this study provided by Pooyandegan Rah Saadat Company. The capnography device was a main wave form of capnography. The FIO2 was changed to target oxygen saturation about 95%. This was measured in the control group with ABG or pulse oximetry and in the intervention group with pulse oximetry. Equal basic settings were employed on both groups and Fast-track extubation was employed for all the patients.

### ASV Mode

Adaptive Support Ventilation (ASV) is an intelligent advanced closed-loop ventilation mode that maintains constant minimum mandatory ventilation based on preset minute volume% and ideal body weight. Depending on patients’ status, this mode provides a range of ventilation support including Pressure Controlled Ventilation (PCV), Synchronized Intermittent Mandatory Ventilation (SIMV), or Pressure Support Ventilation (PSV). Ventilator switches between these forms automatically. ASV adjusts respiratory rate, tidal volume, and inspiratory time continuously and it depends on the mechanism of the lung and the efforts of the patients (26, 27). The controlled settings of this mode included the following items: patients’ ideal body weight (IBW), the value of minute respiratory volume in percentage (Min volume), positive end expiratory pressure (PEEP), fraction of inspired oxygen (Fio2), the level of ventilator sensitivity for the breath of the patient (Trigger), and the maximum pressure that must be applied (cmH2o). The most important settings in ASV are ideal body weight and Minute volume. The ventilator uses the Otis equation for determining the appropriate rate and volume based on measuring patients’ respiratory compliance and resistances during the initial five breaths. By improving the patient situation, ventilator switches from controlled breaths to spontaneous breaths. During the spontaneous breathing, gradually by improving the patient situation, the inspiratory pressure support decreases. Ventilator continuously adapts the settings and supports patients’ needs.

### Training

In spite of published data about capnography, it is not used widely in Iranian clinical practice. For this reason, in order to reduce the staff, fear of its accuracy a 4-hour training class was conducted for ICU staff with clinical work in the pilot phase for one month. In those sessions, the data from the capnography and ABG were compared and the results were presented to them. Based on our data, the Etco2 was usually 2–4 mmHg lower than PaCO2. Thus, this difference was considered in all the settings and interpretation regarding PaCO2.

### Statistical Analysis

Categorical variables were described as frequency rates and percentages, and continuous variables were described using mean ± standard deviation (SD) values. The comparison of demographic characteristics and baseline measures between the two groups herein were done with independent t-test for continuous variables and Chi-square test or Fisher’s exact test (in case of low sample) for categorical variables. The normality of the numeric variables was checked employing Kolmogorov-Smirnov test. The repeated measures analysis of variance (RMANOVA) was used to compare the vital signs and ABG measurements between the groups. Furthermore, we did pairwise comparisons with Sidak post hoc test. The assumption of sphericity was addressed utilizing Mauchly’s test of sphericity. Once the assumption was not satisfied (*p *< 0.05), the Greenhouse-Geiser correction of *p*-value was utilized. To assess the effect of intervention, we used the analysis of covariance (ANCOVA) after controlling for baseline measures and confounders in the main effect model. All the data were analyzed using the Statistical Package for the Social Sciences (SPSS) 21.0 statistical package (Chicago, IL, USA); two-side *p* < 0.05 indicated a statistically significant difference.

## Results

**Figure 2:**
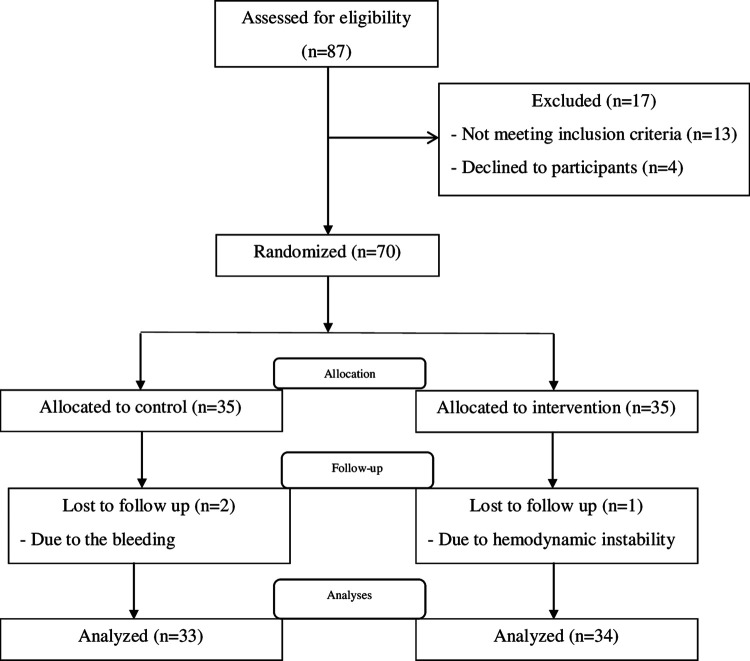
The study flow chart.

A total of 70 patients enrolled in the current study according to the inclusion and exclusion criteria. Three patients were excluded due to hemodynamic instability (1 subject from the intervention group) and bleeding (2 subjects from the control group). Therefore, the analyses were performed in the remaining 67 patients (34 patients in the intervention group and 33 patients in the control group). Figure 2 depicts the patients’ flowchart of the study.

The mean ± SD ages of patients in the intervention and control groups were 58.1 ± 9.6 and 60.8 ± 9.3 years, respectively. There were no significant differences between the groups in terms of age (*p *= 0.244). In terms of gender, 19 participants (55.9%) in the intervention group and 22 (66.7%) in the control group were male (*p *= 0.365). Baseline demographic and clinical characteristics of the participants in the two groups are presented in Table 1. Based on our findings, there were no significant differences between the groups regarding the demographic characteristics and baseline ABG measurements (*p > 0.05*). However, in terms of intraoperative parameters, the duration of the surgery (based minutes) in the control group was significantly lower than that in the intervention group (235.7 ± 49.2 min vs. 259 ± 39.8 min, *p *= 0.035).

**Table 1:** Baseline demographic and clinical characteristics of the participants in two groups of study.

Variables	Intervention group (*n* = 34)	Control group (*n* = 33)	*p*-value
Demographic characteristics			
Age (mean ± SD)	58.12 ± 9.66	60.85 ± 9.32	0.244
Gender (male, %)	19 (55.9)	22 (66.7)	0.365
Body mass index (mean ± SD)	27.93 ± 4.38	27.81 ± 4.78	0.908
Smoking (yes, %)	1 (2.9)	6 (18.2)	0.054
Opium history (yes, %)	5 (14.7)	1 (3)	0.197
Comorbidities			
Renal diseases (yes, %)	3 (8.8)	1 (3)	0.614
Diabetes mellitus (yes, %)	15 (44.1)	16 (48.5)	0.808
Hypertension (yes, %)	22 (64.7)	19 (57.6)	0.549
Clinical characteristics			
EF (mean ± SD)	47.21 ± 7.09	48.94 ± 5.41	0.226
Pump time, min (mean ± SD)	63.32 ± 25.69	57.33 ± 27.94	0.364
Intraoperative parameters			
Type of operation (CABG, %)	19 (55.9)	22 (66.7)	0.365
DO, min (mean ± SD)	259 ± 39.87	235.76 ± 49.2	0.035^*^
Arterial blood gas (ABG) measurements			
Blood PH (mean ± SD)	7.36 ± 0.04	7.36 ± 0.04	0.804
PaCO2, mm Hg (mean ± SD)	37 ± 4.30	36.18 ± 4.48	0.449
PaO2, mm Hg (mean ± SD)	114.41 ± 45.30	10.3.18 ± 32.91	0.250
SpO2, % (mean ± SD)	97.41 ± 2.13	96.82 ± 2.06	0.252
BE, mm Hg (mean ± SD)	−4.08 ± 2.24	−4.50 ± 2.15	0.436
HCO3, mEq/L (mean ± SD)	21.19 ± 1.99	19.90 ± 4.68	0.147

EF, Ejection fraction; DO, Duration of operation; PaCO2, partial pressure of carbon dioxide; PaO2, partial pressure of oxygen; SpO2, Oxygen saturation; BE, Base excess; HCO3, Bicarbonate *<0.05 was considered statistically significant.

Comparisons of hemodynamic parameters and vital signs of the patients on different times between the two groups and within group are respectively presented in Tables 2 and 3. There were significant differences between the intervention and control groups concerning tidal volume (VT) *p *= 0.016, inspiratory pressure (Pi) *p* = 0.006, and fraction of inspired oxygen (Fio2) *p *= 0.002. Nevertheless, the differences of other characteristics between the two groups were not significant (*p *> 0.05).

**Table 2:** Comparison of ventilator and respiratory in three different times between the intervention and control groups.

Parameters	Groups	Baseline	Trigger time	Extubation time	*p*-value^**^	*p*-value^***^
VT, min	Intervention	100	82.35 ± 4.96	77.21 ± 6.87	<0.001	0.016
	Control	100.61 ± 2.07	86.97 ± 6.72	78.64 ± 5.89	<0.001	
	*p*-value^*^	0.093	0.002	0.365		
Pi, cmH2O	Intervention	12.62 ± 1.64	10.88 ± 1.75	8.35 ± 1.68	<0.001	0.006
	Control	12.85 ± 1.50	11.61 ± 1.54	10.03 ± 1.57	<0.001	
	*p*-value^*^	0.603	0.078	<0.001		
Fio2, %	Intervention	52.94 ± 6.29	40.44 ± 1.89	40	<0.001	0.002
	Control	58.94 ± 10.05	43.03 ± 5.98	41.67 ± 4.44	<0.001	
	*p*-value^*^	0.005	0.019	0.033		
PEEP, cmH2O	Intervention	4.94 ± 0.34	4.94 ± 0.34	4.91 ± 0.37	0.325	0.679
	Control	5	5	4.88 ± 0.54	0.211	
	*p*-value^*^	0.328	0.326	0.774		
Flow total	Intervention	12.32 ± 1.06	13.26 ± 2.72	16.21 ± 2.64	<0.001	0.785
	Control	12.30 ± 0.84	13.55 ± 2.26	15.64 ± 2.57	<0.001	
	*p*-value^*^	0.931	0.648	0.375		
Flow control	Intervention	12.32 ± 1.06	4.32 ± 3.28	–	<0.001	0.054
	Control	12.30 ± 0.84	6.18 ± 3.72	–	<0.001	
	*p*-value^*^	0.931	0.034	–		
TVE	Intervention	545.5 ± 122.2	459.7 ± 111.7	487.5 ± 119.15	0.231	0.322
	Control	500.1 ± 91.61	488.2 ± 98.91	482.03 ± 91.8	0.274	
	*p*-value^*^	0.090	0.309	0.833		

*VT, Tidal volume; Pi, Inspiratory pressure; Fio2, Fraction of inspired oxygen; PEEP, Positive end-expiratory pressure; TVE, Total expiratory volume, p < 0.05 was considered statistically significant*.

*Independent t-test between two groups.

**Time-interaction within group based on RMANOVA.

***Time-interaction between two groups.

**Table 3:** Comparison of vital signs of patients on different times between the intervention and control groups.

Parameters	Groups	Baseline	Trigger time	Extubation time	Post extubation	*p*-value^**^	*p*-value^***^
Heart rate, bpm	Intervention	83 ± 12.31	86.47 ± 11.85	89.47 ± 9.45	88.03 ± 8.49	0.002	0.380
	Control	86.24 ± 12.71	87.48 ± 13.45	91.36 ± 12.43	90.7 ± 12.51	0.019	
	^*^p-value	0.293	0.744	0.485	0.310		
RR, per/min	Intervention	12.32 ± 1.06	13.06 ± 2.04	16.21 ± 2.64	16.85 ± 2.24	<0.001	0.755
	Control	12.24 ± 0.83	13.61 ± 2.20	16.03 ± 2.69	16.97 ± 1.81	<0.001	
	^*^p-value	0.730	0.296	0.789	0.816		
Systolic BP, mm Hg	Intervention	119.24 ± 15.7	126.06 ± 12.55	126.26 ± 13.61	119.29 ± 11.44	0.010	0.653
	Control	113.03 ± 23.7	127.76 ± 18.17	125.06 ± 12.03	120.33 ± 18.22	0.006	
	^*^p-value	0.210	0.657	0.703	0.780		
Diastolic BP, mm Hg	Intervention	61.38 ± 10.92	67.88 ± 8.37	67.56 ± 7.89	65.94 ± 9.76	0.004	0.736
	Control	61.79 ± 12.65	68.79 ± 9.76	68.76 ± 9.13	65.70 ± 11.43	0.011	
	^*^p-value	0.889	0.685	0.567	0.925		
MAP, mm Hg	Intervention	79 ± 12.01	85.74 ± 8.83	85.32 ± 8.09	81.94 ± 9.79	0.011	0.851
	Control	76.09 ± 13.13	85.67 ± 11.34	84.82 ± 10.44	84.09 ± 12.23	0.001	
	^*^p-value	0.347	0.978	0.825	0.429		
SpO2, %	Intervention	98.2 ± 1.68	97.41 ± 1.32	97.55 ± 1.76	97.29 ± 1.36	0.034	0.169
	Control	98.87 ± 1.51	97.42 ± 1.56	96.87 ± 1.86	96.81 ± 1.94	0.013	
	^*^p-value	0.407	0.972	0.130	0.249		

*RR, Respiratory rate; BP, Blood pressure; MAP, Mean arterial pressure; SpO2, Oxygen saturation.*

*Independent t-test between two groups.

**Time-interaction within group based on RMANOVA.

***Time-interaction between two groups.

**Figure 3:**
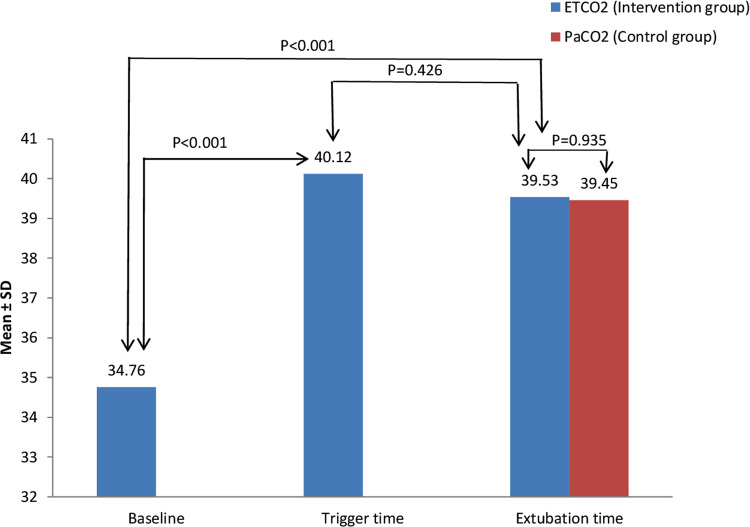
ETCO2 level distribution in the intervention group compare with PaCO2 in the control group at extubation time

Figure 3 shows ETCO2 level distribution of patients in intervention group compare with the PaCO2 in control group at extubation time. The mean of ETCO2 in the intervention group was compared to the PaCO2 in the control group at extubation time. The data indicated no statistically significant differences between them (39.5 ± 3.0 vs. 39.4 ± 4.3, 95% CI, −1.7–1.8, *p *= 0.935).

The mean duration of MV was 212.2 ± 80.6 (min) and 342.7 ± 110.7 (min) in the intervention and control groups, respectively (*p *< 0.001). FTE (MV duration <6 hours) was observed in 33 (97.1%) of the patients in the intervention group and 18 (54.5%) of the patients in the control group. The intervention group was of 4.8 (95% CI, 1.4–15.5; *p *= 0.008) times more chance than the control group from FTE perspective. However, the differences in the length of stay in ICU between the two groups was not significant (*p *= 0.219). The number of alarms (*p *< 0.001) and manual ventilator setting changes (*p *= 0.027) were significantly lower in the intervention group. In addition, no significant differences were observed in compliance between the groups (*p *= 0.289). Meanwhile, the differences in the mean of airway resistant between the two groups was significant (*p *= 0.045). Comparison of extubation time, length of ICU stays, airway resistance, and compliance are listed in Tables 4 and 5.

**Table 4:** Comparison of airway resistance and compliance in different times between the intervention and control groups.

Parameters	Groups	Baseline	Trigger time	Extubation time	*p*-value^**^	*p*-value^***^
Resistance (cm H2O/L/sec)	Intervention	8.03 ± 1.99	8.18 ± 1.51	7.38 ± 1.59	0.025	0.049
	Control	8.64 ± 1.93	8.3 ± 1.81	8.76 ± 1.73	0.362	
	*p*-value^*^	0.210	0.757	0.001		
Compliance (L/cm H2O)	Intervention	50.62 ± 16.01	49 ± 14.08	49.03 ± 14.77	0.702	0.289
	Control	53.64 ± 16.01	51.39 ± 15.74	53.18 ± 14.19	0.677	
	*p*-value^*^	0.435	0.514	0.244		

*Independent t-test between two groups.

**Time-interaction within group based on RMANOVA.

***Time-interaction between two groups, Sidak post hoc tests shows significant differences between resistance in trigger and extubation time (p = 0.005).

**Table 5:** Comparison of extubation time and length of ICU stay between the intervention and control groups.

Parameters	Intervention (*n* = 34)	Control (*n* = 33)	*p*-value
Alarm (mean ± SD), number	16.38 ± 3.82	19.75 ± 3.45	<0.001^*^
Manipulations (mean ± SD), number	6.20 ± 2.08	7.72 ± 3.29	0.027
Time to extubation, min	212.20 ± 80.61	342.72 ± 110.73	<0.001
Length of ICU stay, day	1.96 ± 0.19	2.10 ± 0.55	0.219
Early extubation (<6 h)	33 (97.1%)	18 (54.5%)	<0.001^**^

*Independent t-test.

**Fisher’s exact test.

## Discussion

In this study, the use of non-invasive monitoring including capnography and pulse oximetry accelerated the weaning process and increased the number of FTE in intervention group. According to the results of the present study, in terms of vital signs and the amount of arterial oxygen saturation (SpO2), there were no significant differences between the intervention and control groups. However, there was a significant difference between the two groups regarding tidal volume (VT), inspiratory pressure (Pi), and fraction of inspired oxygen (Fio2). In the current study, the number of manual setting changes of ventilator and alarms in the intervention group significantly decreased compared to the control group. The reduction in both alarm and manual setting changes is of great importance. In fact, the increase in the number of alarms may lead to alarm fatigue, which is an important concern for patients’ safety (28–30).

In addition, our data revealed no significant differences between the values of PaCO2 and ETCO2 at the weaning time; however, we observed a statistically significant linear relationship between them (*p *< 0.001). PaCO2 and ETCO2 had a strongly positive correlation (*r* = 0.918), meaning that these variables tend to increase together. In other words, ETCO2 could be used instead of PaCO2 for monitoring. In this regard, the results of the present study are consistent with those of the studies conducted by Taghizadeh et al. (10), Aminiahidashti et al. (31), McSwain et al. (32), Moses et al. (33), and Garcia et al. (34). They all reported the same results in terms of the correlation between ETCO2 and PaCO2 and suggested that capnography could be employed as a non- invasive method, and it is a surrogate for PaCO2 which if high, signals a respiratory acidosis or if low, a respiratory alkalosis or when combined with pH and serum bicarbonate, a compensatory mechanism. In contrast to our results, certain studies have reported that the correlation between these variables is not sufficient (35–37). A pilot study by Drew et al. (38), in which they used non-invasive methods during the weaning process, exhibited that the hypothesis that capnography would allow more rapid weaning from MV, and requires fewer ABGs during the process, was not true. Moreover, in a study by Casati et al. (39), the role of capnography was reported to be inappropriate in patients with spontaneous breathing. This contradiction may be attributed to the different research methods or different populations. For example, some studies have generally not included a comprehensive statistical analysis accounting for the differences in physiologic dead space ventilation and the resulting gradient between ETCO2 and PaCO2. In addition, some other studies have been reviewed in patients with critical conditions.

The results of this work revealed that the use of capnography and pulse oximetry accelerated the weaning process in the patients undergoing CABG surgery. FTE is found to be the best practice for management of cardiac surgery patients, which improves the recovery and reduces the complications (40). In this study 97.1% of the intervention group and 54.5% of the control group had FTE, which was reported 49.5% FTE in a study by Akhtar et al. (5). Our results showed that the use of capnography could improve the FTE by the identification of hypercapnic episodes during the weaning process. In some centers, due to the fear of hypercapnic episodes, the FTE is delayed. Hence, using ETCO2 with continuous monitoring can reduce this fear. On the other hand, one of the uses of capnography is the discovery of hypercapnic episodes (41).

In some studies, changes in ETCO2 was observed from breath to breath and from the controlled to the spontaneous breathing (6). Therefore, the momentary monitoring is not reliable and the ETCO2 should be monitored continuously during the weaning process. Due to the importance of FTE in cardiac surgery, various changes are made to MV settings in a short time. Due to some restrictions such as limited access to ABG analyzers in developing countries and the fact that ABG method is an invasive method, a non-invasive monitor, which is easily accessible, inexpensive, and accurate, is very useful. The available data showed that both capnography and pulse oximetry have the above-mentioned features.

Despite the availability of capnography in many ICUs, it is not frequently used in the routine practice. One of the reasons might be the medical staff’s fear of the risks and the proper linkage between the PaCO2 and ETCO2 (42, 43). As mentioned in the method section, we had a 4-hour class with a pilot clinical phase for assuring the staff of the accuracy of Etco2. This increased the trust of ICU staff.

The principal strength of this study is being conducted in a real-world setting. All the patients were screened by a pulmonologist and the medical staffs in our center were trained. The limitation of this study is the sample size which was small; therefore, it was not possible to determine the cutting point for ETCO2. Accordingly, a larger sample size study is recommended to determine the cutting point.

## Conclusion

Using the non-invasive monitors including capnography and pulse oximetry enhances the rate of FTE in cardiac surgery. It is also a safe and correct monitor and could be a good alternative for ABG in this population. Nevertheless, the fact that in some conditions the relationship between ETCO2 and PaCO2 was not well described primarily necessitates the determination of a capnography on which patients could rely. Precautions should be taken and other respiratory parameters, including tidal volume and respiratory rate, should be taken into consideration. In addition, further studies are required with larger sample sizes in different disease status and populations for the assessment of the accuracy of our findings.

## Data Availability

The original contributions presented in the study are included in the article/Supplementary Material, further inquiries can be directed to the corresponding author/s.
